# Coronary heart disease in primary care: accuracy of medical history and physical findings in patients with chest pain – a study protocol for a systematic review with individual patient data

**DOI:** 10.1186/1471-2296-13-81

**Published:** 2012-08-09

**Authors:** Jörg Haasenritter, Marc Aerts, Stefan Bösner, Frank Buntinx, Bernard Burnand, Lilli Herzig, J André Knottnerus, Girma Minalu, Staffan Nilsson, Walter Renier, Carol Sox, Harold Sox, Norbert Donner-Banzhoff

**Affiliations:** 1 Department of General Practice/ Family Medicine, University of Marburg, Marburg, D-35032, Germany; 2 Universities of Leuven and Hasselt, Interuniversity Institute for Biostatistics and Bioinformatics, Leuven, Belgium; 3 Department of General Practice, Katholieke Universities Leuven, Leuven, Belgium; 4 Lausanne University Hospital, Lausanne, Switzerland; 5 Institute of General Medicine, University of Lausanne, Lausanne, Switzerland; 6 Department of General Practice, University of Maastricht, Maastricht, Netherlands; 7 Department of Medical and Health Sciences, General Practice, Linköping University, Linköping, Sweden; 8 The Dartmouth Institute for Health Policy and Clinical Practice/ , Dartmouth Medical School, Lebanon, USA

**Keywords:** MeSH, Chest pain, Myocardial ischemia, Medical history taking, Sensitivity and specificity, Primary health care

## Abstract

**Background:**

Chest pain is a common complaint in primary care, with coronary heart disease (CHD) being the most concerning of many potential causes. Systematic reviews on the sensitivity and specificity of symptoms and signs summarize the evidence about which of them are most useful in making a diagnosis. Previous meta-analyses are dominated by studies of patients referred to specialists. Moreover, as the analysis is typically based on study-level data, the statistical analyses in these reviews are limited while meta-analyses based on individual patient data can provide additional information. Our patient-level meta-analysis has three unique aims. First, we strive to determine the diagnostic accuracy of symptoms and signs for myocardial ischemia in primary care. Second, we investigate associations between study- or patient-level characteristics and measures of diagnostic accuracy. Third, we aim to validate existing clinical prediction rules for diagnosing myocardial ischemia in primary care. This article describes the methods of our study and six prospective studies of primary care patients with chest pain. Later articles will describe the main results.

**Methods/Design:**

We will conduct a systematic review and IPD meta-analysis of studies evaluating the diagnostic accuracy of symptoms and signs for diagnosing coronary heart disease in primary care. We will perform bivariate analyses to determine the sensitivity, specificity and likelihood ratios of individual symptoms and signs and multivariate analyses to explore the diagnostic value of an optimal combination of all symptoms and signs based on all data of all studies. We will validate existing clinical prediction rules from each of the included studies by calculating measures of diagnostic accuracy separately by study.

**Discussion:**

Our study will face several methodological challenges. First, the number of studies will be limited. Second, the investigators of original studies defined some outcomes and predictors differently. Third, the studies did not collect the same standard clinical data set. Fourth, missing data, varying from partly missing to fully missing, will have to be dealt with.

Despite these limitations, we aim to summarize the available evidence regarding the diagnostic accuracy of symptoms and signs for diagnosing CHD in patients presenting with chest pain in primary care.

**Review registration:**

Centre for Reviews and Dissemination (University of York): *CRD42011001170*

## Background

Chest pain is a frequent complaint in many health care settings. In primary care 0.7% to 2.7% of patient encounters are due to chest pain [1-3]. However, the prevalence of serious cardiac disease in these patients, e.g., chronic stable coronary heart disease (CHD) or acute coronary syndrome (ACS), is low. In unselected patients presenting with chest pain in primary care, the overall prevalence of coronary heart disease is between 12.8 and 14.6% [2,3]. In the majority of patients, the underlying etiology is musculoskeletal, esophageal, respiratory, psychological, or is unknown.

Primary care physicians (PCP) face several challenges in diagnosing CHD. In primary care, patients with CHD often present in the early stages of their disease, often with uncharacteristic clinical findings that make the separation from other etiologies difficult. PCPs must reliably identify serious cardiac disease while also protecting patients from unnecessary testing and hospital admissions. They must rely on the history, physical findings, and their accumulated knowledge of an individual patient to determine the clinical probability of CHD and decide whether testing, specialist referral or hospital admission is indicated. Tests ( i.e. troponin levels, and the electrocardiogram) lack sensitivity in the early stages of myocardial infarction (MI) and cannot exclude acute ischemia in patients with a high clinical probability. Lastly, the optimal early evaluation of possible CHD uses the patient’s clinical probability in order to decide on the value of further testing and to interpret test results using probabilistic reasoning, a form of thinking that many physicians do not use [4].

The accuracy of medical history and physical examination for CHD has been the subject of previous meta-analyses. Mant et al.. studied the diagnostic value of symptoms and signs for ACS and myocardial infarction (MI) in studies published until 1999 [5]. Bruyninckx et al. focused on the value of 10 pre-specified clinical symptoms and signs in diagnosing ACS and MI [6]. Chun and McGee did not restrict their research question to pre-specified symptoms. Target diseases were stable CHD, ACS, and MI [7]. Their search was conducted in 2003.

The studies included in these reviews have important limitations, especially for application to the primary care setting. Nearly all were conducted in emergency departments of hospitals, or secondary and tertiary care. The respective settings differ with regard to the prevalence and clinical presentation of CHD or other serious conditions. Sox and colleagues showed that varying prevalence of CHD in primary and secondary care resulted in diverging predictive values of a clinical prediction rule [8]. Additionally, several authors have supposed that sensitivity and specificity of medical tests also vary across different settings [9-11]. Thus, results of diagnostic accuracy studies conducted in other settings should not be assumed to apply to office-based primary care.

Furthermore, the authors of these reviews synthesized the results using aggregate data from each study, such as the 2 by 2 tables of diagnostic accuracy. In contrast, meta-analyses using individual patient data (IPD) consider the whole information about each patient that is available in the primary studies. This increases the scope of possible statistical analyses. For example, IPD meta-analysis allows investigation of the relationship between patient-level characteristics like sex and age and the diagnostic accuracy of a test [12,13]. Single symptoms and signs are rarely sufficient to reliably diagnose CHD. This problem may be overcome by developing a prediction rule that combines several symptoms, signs, and other patient characteristics like sex, age and coronary risk factors. A meta-analysis using individual patient data allows the construction of such a prediction rule as well as the validation of existing clinical prediction rules.

### Objectives of the study

In order to provide PCPs with evidence that applies to their clinical setting, we will perform a systematic review and meta-analysis using individual patient data. We will include only studies conducted in office-based primary care. The aims of this review are:

· to determine the diagnostic accuracy of symptoms and signs for myocardial ischemia in primary care patients

· to explore possible associations between study- or patient-level characteristics and measures of diagnostic accuracy.

· to validate existing prediction rules for diagnosing myocardial ischemia in primary care.

The aim of this preliminary report is to describe the methods of our study and the study populations that comprise the meta-analysis.

## Methods/Design

### Study inclusion criteria

The studies to be included must fulfill the following criteria:

#### Patients

We will include studies recruiting unselected adult patients presenting with chest pain in office-based primary care practice. Studies are not eligible if the patients have been recruited in emergency departments of hospitals, by paramedics, or if the patients have been pre-selected by PCPs or other health professionals based on the likelihood of an underlying CHD. If patients of all age groups are included in the original study we will exclude patients aged < 18 years in the analysis.

#### *Index tests*

The diagnostic tests under evaluation will include items of history and physical examination like symptoms, signs, age, sex, coronary risk factors. We will not exclude studies or discard individual items because the studies defined the items differently or used different wording of the questions in the standard data set.

#### *Target disease/ reference standard*

CHD is the reference condition. We will not exclude studies because of differing case definitions (e.g. ACS vs. stable CHD). Coronary angiography is supposed to be the definite reference standard in diagnosing CHD. However, since the likelihood of CHD in most patients presenting in primary care is low, this reference test is too invasive. Knottnerus and colleagues suggest that follow up of the clinical course during an appropriate period is a good alternative. They refer to this design as delayed-type cross-sectional design [14]. However, we will not exclude studies because of differing method for establishing the reference diagnosis (e.g. independent reference panel; diagnosis by the GP that cared for the patient).

#### *Data collection*

We will exclude studies in which the clinical findings were obtained retrospectively from medical records.

### Study identification and study selection

We will perform a computerized search in MEDLINE (National Library of Medicine), and EMBASE (Excerpta Medica). Terms identifying chest pain will be used along with terms to identify studies conducted in primary care. Search strategies will include subject headings (MeSH, Embtree) as well as free-text terms (Table 1). Furthermore, will perform a hand search in the online published abstracts of the annual meetings of the North American Primary Care Research Group and the European General Practice Research Network. Additionally, we will check the reference lists of all relevant articles. We will ask authors of relevant articles if they are aware of studies which are unpublished, ongoing, or which we have not identified until now. We invite readers who know of such studies to contact us.

**Table 1:** Search terms (PubMed)

			
**Searching for chest pain in title/abstracts**	chest[TIAB]		pain[TIAB]
	OR		OR
	thoracic[TIAB]		pains[TIAB]
	OR		OR
	thorax[TIAB]		discomfort[TIAB]
		AND	OR
			complaint*[TIAB]
			OR
			distress*[TIAB]
			OR
			pressure*[TIAB]
			OR
			numbness[TIAB]
OR			
**Searching for chest pain in MeSH Terms**	"chest pain"[MeSH]		
**AND**			
**Searching for Primary Care in title/ abstract**	"general practitioner”[TIAB]		
	“general practitioners”[TIAB]		
	“general practice”[TIAB]		OR
	“family practice”[TIAB]		
	“family practitioners[TIAB]		
	“family practitioner”[TIAB]		
	“family medicine”[TIAB]		
	“family physician”[TIAB]		
	“family physicians”[TIAB]		
	“family doctor”[TIAB]		
	“family doctors”[TIAB]		
	“primary care”[TIAB]		
OR			
**Journales relevant for Primary Care**	"BMC Fam Pract"[TA]		
	"Fam Pract"[TA]		
	"J Fam Pract"[TA]		
	"Fam Pract Res J"[TA]		
	"J Am Board Fam Pract"[TA]		
	"Br j gen pract"[TA]		
	“J R Coll Gen Pract” [TA]		
	“J Coll Gen Pract” [TA]		
	“J Coll Gen Pract Res Newsl”[TA]		OR
	"Can fam physician"[TA]		
	"Ann Fam Med"[TA]		
	"Aust fam physician"[TA]		
	"Scand J Prim Health Care"[TA]		
	"Eur J Gen Pract"[TA]		
	“J Gen Intern Med”[TA]		
	"Arch Fam Med"[TA]		
	"Aten Primaria"[TA]		
OR			
**Searching for Primary Care in the affiliation of the author**	“general practice” [AD]		
	“family practice*” [AD]		
	“family medicine”[AD]		OR
	“primary care” [AD]		
	community [AD]		
**Searching for Primary care in MeSH**	"Family Practice"[MeSh]		
	"Physicians, Family"[MeSh]		OR
	"Primary Health Care"[MeSh]		

Limits: NOT: Editorial, Addresses, Bibliography, Biography, Case Reports, Comment, Dictionary, Directory, Festschrift, Government Publications, Guideline, Historical Article, In Vitro, Interactive Tutorial, Interview, Legal Cases, Legislation, Patient Education Handout, Portraits, Webcasts.

### Study quality assessment

At study level we will extract information on methodological characteristics of the studies, such as inclusion criteria, patient recruitment, data collection, and reference standard from publications. Data not retrievable from published reports will be requested from original investigators. Two reviewers, who have not been involved in the conduct of any of the primary studies, will independently assess the internal validity and methodological quality of each study using the quality assessment tool for diagnostic accuracy studies (QUADAS) [15,16]. They will resolve disagreements by discussing their findings.

### Outcome and index tests

Each study in our final data set will contain data on patient identifier code, patient’s age and gender, PCP identifier code, data on absence or presence of at least one symptom or sign, and results on the final diagnosis. The diagnostic outcome variable will be any manifestation of CHD. As index tests we will consider each item of the medical history or clinical examination. However, we cannot expect that all studies gathered data on the same symptoms and signs or that they used the same wording, definition, operationalization, or coding of the data. Because the original questionnaires or case report forms had been written in different languages, we will translate the variables used in the individual studies into English and will create a synopsis showing the names, definitions and categories of all variables. Using this synopsis we will identify any symptom and sign that was collected in at least two studies and that should therefore be included in the analysis. Based on the studies we have identified so far following variables will be included in the analysis:

Age

Sex

Context of consultation

Did the patient require a home visit?

Did the PCP know the patient?

Was the encounter an emergency/ an urgent visit?

Was chest pain the main complaint?

Was CP present during consultation?

Had the patient experienced CP before?

PCPs’/ Patients’ concern

Did the GP assumed something serious?

Probability of CHD rated by PCP

Was the patient anxious?

Did the patient think the pain is related to the heart?

Pain characteristics - localization

Retrosternal CP

Precordial CP

Left-sided CP

Right-sided CP

Pain characteristics – radiation

Band-shaped radiation of pain

CP radiating to left shoulder/arm

CP radiating to right shoulder/arm

CP radiating to neck, jaw, bottom side of face

CP radiating to abdomen

Pain characteristics intensity

Intensity of CP

Pain characteristics – quality/ word descriptors

Stabbing pain

Oppressive pain

Burning pain

Dull pain

Pain characteristics – duration and course

Time since first occurrence of the pain

Continuous pain

Duration of a typical pain episode

Frequency of CP

Clinical Course of pain

Pain characteristics - classification

Typical angina

Unspecific CP

Pain depend on/ provoked by / related to

CP related to breathing

CP related to movement

CP related to swallow

CP related to ingestion

CP related to effort/ exercise

CP related to cough

CP related to body position

Relief after administration of NTG

Relief after administration of antacid

Additional complaints/associated symptoms

Fever

Cough

Dyspnea

Sweating

Paleness

Nausea

Signs of a cold/ respiratory infect

Sputum

Reduced state of consciousness

Physical examination

Heart rate

Blood pressure

Arrhythmia

Pulmonary auscultation

Cardiac auscultation

Pain reproducible by palpation

Medical history – coronary risk factors, previous cardiac events

History of dyslipidemia/ hyperlipidemia

History of diabetes (diabetes mellitus)

Family history of myocardial infarction

History of hypertension

Smoking

History of myocardial infarction

History of known CHD

Number of cardiovascular risk factors

(*PCP* primary care physician, *CP* chest pain, *CHD* coronary heart disease)

### Data management

Using the synopsis of the variables we will recode the original data sets. We will then check the recoded data sets whether values seem to be plausible, realistic, consistent, and at least similar to the results published by the researchers. Additionally, the individual investigators of the each of the original studies will validate the results of the translation and recoding process.

Cases aged < 18 years and cases with missing values in the diagnostic outcome variable will be excluded. If results on individual index tests are available on study level but are missing on individual patient level we will consider this as missing at random and impute the missing values. According to the well-established approach of multiple imputation we will create five imputed data sets for each original study [17,18] and merge them into five imputed meta data sets. Translation and recoding will be a potential source of between-study heterogeneity. If substantial between-studies heterogeneity will occur in the analyses of single symptoms and signs, translation and matching of the respective variables will have to be discussed by the study team.

### Data analysis

Primary objectives of the statistical analyses are to investigate the diagnostic accuracy of symptoms and signs and to explore possible associations between study- or patient-level characteristics and measures of diagnostic accuracy. In order to achieve these aims we will use different approaches.

In the *bivariate* analysis we will investigate the diagnostic accuracy of single symptoms and signs. In a first study-specific step we will calculate values of sensitivity, specificity and likelihood ratios for the single index tests within the individual studies. All statistical models in this step will be formulated as logistic regression models, with test status as response, and the disease status as explanatory variable [19]. To deal with sparsity (small number of cases in certain combinations of CHD status and symptoms and signs), we will apply Firth’s method to correct for small sample bias [20]. We will combine the results of the imputed data sets within the individual studies using recommended techniques of multiple imputation [17,18]. We will plot the results for each clinical feature in forest plots. It is possible to extend the model to evaluate the impact of age and gender within the individual studies. However, the ultimate goal of this approach is to pool the results for a single symptom and sign across studies while accounting for individual patient (age and gender) and study characteristics. The feasibility of the bivariate random effects meta-analysis framework that Riley and colleagues recommended for this task largely depends on the number of studies [12]. Since the studies do not provide data on the all index tests, the number of studies considered in the individual analyses will vary between 2 and n, where n is the number of all included studies.

In the *multivariate* approach we will determine optimal combinations of symptoms and signs. Similar to the bivariate analysis we will use a stepwise approach. In a first step, the *study-specific multivariate analysis*, we will determine optimal combinations of symptoms and signs within the individual studies. In order to achieve that aim we will use a random forest algorithm to identify the most important index tests in each study. The random forest algorithm is a powerful data-driven method for identifying important variables [21]. Next, we will fit a logistic regression model with all index tests selected in this way and their two way interaction terms. We will perform these steps separately for each imputed data set of each original study. All index tests which will be significant (α = 5%) in at least one of the study-specific imputed data sets will be included in a candidate list. We will combine the results of the imputed data sets within each individual study according to the inferential methodology of multiple imputation [17,18]. As a result of this step we will identify study-specific models which only include symptoms and signs that are independently associated with the presence of CHD. These study-specific analyses will provide important explorative insights in the heterogeneity between different studies. We expect differences in study design, slightly varying definitions of variables and varying population characteristics to play a role here. Results will guide the model building process for the next step, the *multivariate meta-analysis.* In this approach we will merge the study-specific imputed data sets to five imputed meta data sets. We will enter all index tests included in the candidate list into the model and fit the logistic regression model (1) to each imputed meta data set:

(1) $$\begin{array}{l} \mathrm{Logit}\;\left(P\left(Y_{\mathrm{ij}}=1\right)\right)=\beta_{0\;i\;}+\sum_{k=1}^{P}\beta_{k}X_{\mathrm{kij}}{\text{*I}}_{\text{ki}}\;,\\ \;i=1,\;2,\ldots ,n\text{;}\;j=1,2,\ldots ,n_{i} \end{array}$$

where Y_*ij*_ is the outcome variable for patient *j* in study *i* (0 indicates that CHD is absent, while 1 indicates that CHD is present), *X*_*k*_ represents the result of the *k*th index test, *β*_0*i*_ is the study-specific intercept for the *i*th study, *β*_*k*_ is the coefficient for the index test *X*_*k*_, I_ki_ is the study indicator for the *k*th index test (1 if the test result is available in individual study *i*, or 0 if the test result is not available in individual study *i*), *P* is the number of index tests, *n*_*i*_ is the number of patients in study *i*, and n is the number of studies. Study-specific intercepts will account for heterogeneity across studies and for the fact that the individual studies will contribute data on different sets of index tests. However, model (1) assumes that the effect of a single index test is fixed across studies. A random effects model is possible, but more complex and might be not feasible if the number of studies is small. The multivariate meta-analysis will result in a clinical prediction rule with optimal diagnostic accuracy characteristics. The unique linear combination of selected symptoms and signs with their estimated regression coefficients from the final model defines a “clinical chest pain score”. If the score exceeds a threshold value, the patient is labeled CHD positive; if the score is less than a threshold value, the patient is labeled CHD negative. Such a prediction rule is “personalized” on the one hand and applicable to individuals in a broad community covering several countries or regions and thus exceeding the validity of individual studies on the other hand.

All statistical models considered in the multivariate approach will be formulated as logistic regression models, with CHD as response, and the symptoms and signs as explanatory variables. In order to describe the discriminatory power of the study-specific models and the meta model we will calculate the area under the receiver characteristic curve (AUC). In addition, we will calculate the values of sensitivity, specificity and likelihood ratios for different thresholds of the clinical prediction rule.

Since the maximum likelihood (ML) estimates of a logistic regression model are not necessarily maximizing the AUC, we will compare the ML results with the alternative approach of Pepe et al. [22]. In this approach the estimates are directly maximizing the AUC. By definition the “clinical chest pain score” based on the AUC-maximizing estimates will have improved diagnostics accuracy characteristics, but the improvement might be so negligible that this more computationally demanding approach is not worthwhile. We will examine both approaches, and the final choice will depend on the magnitude and the practical relevance of the improvement. Furthermore, we will apply internal validation techniques in order to validate the “clinical chest pain score” from the final model of the multivariate meta-analysis.

A secondary objective of the statistical analysis is the external validation of clinical prediction rules (CPR) aimed to support PCPs in diagnosing myocardial ischemia in patients with chest pain. We are aware of several rules developed or validated in a primary care setting and based on items of the medical history and clinical examination. [8,23-26]. These CPRs used different predictor variables and definitions of myocardial ischemia, e.g. any CHD, or myocardial infarction. If a study provides data on the required predictors of one or more CPR, we will calculate measures of overall discrimination (area under the curve) and diagnostic accuracy (sensitivity, specificity, likelihood ratios) for recommended thresholds within the individual data set. We will present the results separately for each study and CPR. If the number of studies providing data on one single CPR is sufficient we will calculate pooled estimates of sensitivity and specificity across studies using the approach recommended by Riley et al. [12].

### Studies identified to date

To date, we have identified six relevant studies, including about 4000 patients and conducted in five different countries [2,8,23,27-29]. All six studies investigated prospectively the diagnostic accuracy of symptoms and signs for CHD in consecutive series of patients with chest pain in a primary care setting. The number of patients in the studies ranged from 323 to 1249. Each study used a delayed-type reference standard to establish the reference diagnosis. The studies differ in the duration of follow-up and the person who made the reference diagnosis. Study characteristics are summarized in Table 2. 

**Table 2:** Characteristics of studies identified to date

**Characteristic**	**Sox et al.**[8]	**Buntinx et al.**[23]	**Nilsson et al.**[27]	**Verdon et al.**[2]	**Bösner et al.**[28]	**Haasenritter et al.**[29]
Data collection	1982	1988	1998-2000	2001	2005-2006	2009-2010
Country	USA	Belgium	Sweden	Switzerland	Germany	Germany
Setting	66 PCPs at 1 Drop-in clinic	25 PCPs	3 health care centres each served by 4 PCPs	58 PCPs in private practice	74 PCPs in private practice	56 PCPs in private practice
Number of patients	404*	323	554	672	1249	880
Inclusion criteria	Chest pain as presenting complaint, no age limitation (ages were 17 to 81 years; average 41 years)	New episode of chest pain, discomfort or tightness as main or ancillary complaint	New episode of chest pain, discomfort or tightness as presenting complaint; aged 20–79 years; patients were excluded: if acute MI or coronary re-vascularization during the previous year	Chest pain as main or ancillary complaint; age ≥ 16 years	Chest pain as main or ancillary complaint; age ≥ 35 years; excluded: chest pain ≥ 1 one month, or had already been investigated	Chest pain as main or ancillary complaint; age ≥ 35 years; excluded: chest pain ≥ 1 one month, or had already been investigated
		No age limitation (ages were 17 to 81 years;average 41 years)	No age limitation (ages were 1 to 88 years; average 45 years)			
Reference standard	Delayed-type reference standard	Delayed-type reference standard	Delayed-type reference standard	Delayed-type reference standard	Delayed-type reference standard	Delayed-type reference standard
Duration of follow-up	Average time to diagnosis: 2 months (range – to 8 months)	2 weeks to 2 months	3 months	12 months	6 months	6 months
RD established by	2 internist-investigators independently assigned diagnosis.	Treating physicians	Treating physicians	Treating physicians	Independent expert panel (1GP, 1 cardiologist, 1 research fellow)	Independent expert panel (1GP, 1 research fellow)
Prevalence of CHD as cause of chest pain	7.2%	9.6%	11.2%	12.6%	14.4%	10.6%

*RD* reference diagnosis, *MI* myocardial infarction, PCP primary care physician.

*The number of patients is greater than as previously reported [8] because it includes patients excluded in the published study (diagnosis was acute MI, first episode of chest pain).

The investigators of these six studies have agreed to collaborate as the international working group on chest pain in primary care (interchest) and to provide the data from each study to perform a meta-analysis with individual patient data. An initial meeting took place in January 2010. The coordinating center is the Department of General Practice, University of Marburg, Germany. In a series of video conferences, the whole group has discussed major questions about organizing the data, conducting the analysis, and interpreting the findings. We are inviting investigators of future eligible studies to join this collaboration.

## Discussion

Systematic reviews on the accuracy of diagnostic tests with subsequent meta-analysis of the measures of diagnostic accuracy can play an important role in decision making. They allow more precise estimates of sensitivity and specificity. However, the interpretation of the results is not straightforward. A high degree of heterogeneity or between-study variance that is not due to chance variation is a frequent finding in diagnostic accuracy reviews. Investigating the different sources of this heterogeneity is important [30,31]. Heterogeneity can be caused by study-level characteristics like methodological differences in design or conduct of the study (bias), between-study differences in defining test positives (different cut-points), or other design-related characteristics [30,31]. Furthermore, patient-level characteristics can act as modifiers of diagnostic accuracy [12]. For example, data from secondary care suggests that the sensitivity and specificity of the history and physical findings may vary according to patient characteristics like age [32], sex [33,34], or stage of the target condition. While individual studies often lack statistical power to reliably estimate these effect modifications, aggregate data meta-analysis cannot investigate patient-level modifiers of diagnostic efficacy [12,13].

Most individual symptoms and signs are insufficiently discriminative to diagnose CHD. This problem may be overcome by combining several findings into a clinical prediction rule. Several clinical prediction rules (CPR) have been developed to support physicians in diagnosing myocardial ischemia in patients with chest pains [8,23-26]. We can test these rules in the pooled data set of primary care patients with chest pain.

Several methodological problems make it difficult to anticipate the results of our study. The individual studies were conducted over a span of almost thirty years during which the criteria for myocardial infarction changed, largely because of troponin assays becoming available. We expect semantic and cultural differences to compromise the comparability of study variables. Further, given the results of previous systematic reviews [5-7], the likelihood of finding eligible studies, in addition to the ones previously mentioned, is small. Given a relatively small pooled sample and the novelty of our statistical methods, our statistical analyses should be regarded as exploratory. As guideline developers build upon our findings, they must remember these caveats and remind guideline users that medicine is an inexact art. However, further research in this area requires large samples and sophisticated methods for data collection and analysis. The probability of further studies in this area is therefore small.

We hope that the planned systematic review will advance our understanding in the following ways:

a) Clinical knowledge

We aim to report estimates of accuracy for single signs and symptoms and for clusters of signs and symptoms for the diagnosis of myocardial ischemia in unselected patients presenting with chest pain in primary care. In addition, we will investigate the effect of patient characteristics like sex and age on the diagnostic accuracy of signs and symptoms. Furthermore, we will validate clinical prediction rules for the diagnosis of CHD in primary care. Based on these findings we will be able to give recommendations regarding future research including the investigation of diagnostic algorithms based on combinations of findings of the history, physical examination, and technological devices (ECG, point of care blood tests for troponins or other biomarkers).

b) Methodological knowledge

We will explore possible associations between study-level and patient-level covariates on the one hand, and sensitivity and specificity of medical tests on the other hand. Based on the findings, we aim to provide recommendations regarding the design of future diagnostic studies in primary care and the conduct of diagnostic accuracy reviews based on individual patient data.

## Competing interests

The authors declare that they have no competing interests.

## Authors’ contributions

NDB is the principal investigator of the study described in this article. JH coordinates the study. All authors participate in the study design. JH wrote a first draft of manuscript. All other authors commented on this draft and contributed to the final manuscript. All authors read and approved the final manuscript.

## Funding

This study is funded by Federal Ministry of Education and Research (BMBF - grant no. FKZ 01GK0920). The funding source has no involvement in the study.

## Review registration

PROSPERO, Centre for Reviews and Dissemination (University of York): *CRD42011001170*

## Pre-publication history

The pre-publication history for this paper can be accessed here:

http://www.biomedcentral.com/1471-2296/13/81/prepub
